# Strategic Variants of CSP Delivered as SynDNA Vaccines Demonstrate Heterogeneity of Immunogenicity and Protection from *Plasmodium* Infection in a Murine Model

**DOI:** 10.1128/IAI.00728-20

**Published:** 2021-09-16

**Authors:** Sophia M. Reeder, Mamadou A. Bah, Nicholas J. Tursi, Rebekah C. Brooks, Ami Patel, Rianne Esquivel, Alison Eaton, Hugo Jhun, Jacqueline Chu, Kevin Kim, Ziyang Xu, Fidel Zavala, David B. Weiner

**Affiliations:** a The Perelman School of Medicine, University of Pennsylvania, Philadelphia, Pennsylvania, USA; b The Vaccine Center, Wistar Institute, Philadelphia, Pennsylvania, USA; c Department of Molecular Microbiology and Immunology, Malaria Research Institute, Johns Hopkins Bloomberg School of Public Health, Baltimore, Maryland, USA; UC Davis School of Veterinary Medicine

**Keywords:** DNA vaccines, immunization, malaria

## Abstract

Malaria infects millions of people every year, and despite recent advances in controlling disease spread, such as vaccination, it remains a global health concern. The circumsporozoite protein (CSP) has long been acknowledged as a key target in antimalarial immunity. Leveraging the DNA vaccine platform against this formidable pathogen, the following five synthetic DNA vaccines encoding variations of CSP were designed and studied: 3D7, GPI1, ΔGPI, TM, and DD2. Among the single CSP antigen constructs, a range of immunogenicity was observed with ΔGPI generating the most robust immunity. In an intravenous (i.v.) sporozoite challenge, the best protection among vaccinated mice was achieved by ΔGPI, which performed almost as well as the monoclonal antibody 311 (MAb 311) antibody control. Further analyses revealed that ΔGPI develops high-molecular-weight multimers in addition to monomeric CSP. We then compared the immunity generated by ΔGPI versus synDNA mimics for the antimalaria vaccines RTS,S and R21. The anti-CSP antibody responses induced were similar among these three immunogens. T cell responses demonstrated that ΔGPI induced a more focused anti-CSP response. In an infectious mosquito challenge, all three of these constructs generated inhibition of liver-stage infection as well as immunity from blood-stage parasitemia. This study demonstrates that synDNA mimics of complex malaria immunogens can provide substantial protection as can a novel synDNA vaccine ΔGPI.

## INTRODUCTION

While the global incidence of malaria has decreased in the past decade, the infection remains a significant global health concern. In 2018, there were approximately 228 million cases worldwide, of which 93% were in the WHO African region (1). Those cases resulted in 405,000 fatalities, with a high burden in younger populations, as 67% of cases occur in children under 5 years of age (1). Nearly half of the world’s population is at risk for malaria infection (2). Malaria is caused by infection by *Plasmodium* parasites, which are delivered into humans by mosquitos. While five species can cause disease in humans, Plasmodium falciparum remains the most prevalent malaria parasite, accounting for 99.7% of all cases in the WHO African region (1). Symptoms can arise 10 to 15 days after the infective mosquito bite and include fever, headache, and chills, which can progress to severe illness and potentially death (2).

The WHO Global Technical Strategy for Malaria 2016–2030 established a number of goals to be achieved globally by 2030, including (i) reducing malaria case incidence by 90% by 2030, (ii) reducing malaria mortality by at least 90%, and (iii) eliminating malaria in at least 35 countries (2). Malaria elimination is defined as the interruption of local transmission of a specified malaria parasite species in a defined geographical area as a result of deliberate actions (2). Achieving these goals will require varied approaches, including vector control approaches such as insecticide-treated bed nets and indoor spraying with residual pesticides. The inclusion of a vaccine into these techniques has always been of paramount importance, and this importance continues to grow as drug-resistant strains of the parasite emerge (2).

DNA vaccines represent an important platform for the development of antiparasitic vaccines. Although the first generation of anti-circumsporozoite protein (CSP) DNA vaccines was suboptimal (3, 4), recent developments in DNA technology, including genetic modifications; innovative delivery methods such as jet delivery, gene gun delivery (5), and adaptive electroporation (4); coformulation of plasmid-encoded molecular adjuvants (6, 7); as well as more complex antigen designs (6, 7) have dramatically enhanced immune potency in the clinic (5). In this report, we build on these initial important studies in the DNA space, incorporating newer delivery technology and immunogen designs.

RTS,S/AS01 (RTS,S), one of the longest studied candidate vaccines (developed by GSK over decades) is focused on generating immunity to CSP, the circumsporozoite protein of Plasmodium falciparum. A phase 3 trial that spanned 5 years demonstrated that children aged 5 to 17 months who received 4 doses of RTS,S had a 39% reduction in malaria cases and a 29% reduction in severe malaria cases over 4 years of follow-up (8). After positive recommendations by WHO advisory boards, three countries, Ghana, Kenya, and Malawi, began introducing RTS,S in 2019 (2). In the coming years, the Malaria Vaccine Implementation Programme (MVIP) will assess the feasibility of administering the recommended 4 doses of the vaccine in children, the potential role in reducing childhood death, and the safety of RTS,S in the context of routine use (9) as well as in specific important subpopulations.

An important advance in malaria vaccines is the development of a novel polyvalent immunogen R21, a recent antimalaria vaccine candidate, which builds on the knowledge gained from RTS,S. In contrast to RTS,S, R21 delivers a higher ratio of CSP to the hepatitis B surface antigen (HBsAg), which is in a 1 to 1 ratio as opposed to the 1 to 4 ratio of RTS,S (5). Thus far, R21 has proven to be well tolerated and immunogenic in early trials as well as protective in a controlled human malaria infection study (CHMI) (10, 11) and has shown efficacy in children in Burkina Faso (12). The correlates of immunity, which have emerged from these malaria vaccines, support the importance of anti-CSP antibodies primarily but also suggest a role for T cells for imparting protection from *Plasmodium* infection (8, 10, 11, 13–16). The role of anti-*Plasmodium* T cells in vaccination has been highlighted by attenuated sporozoite vaccines (17).

The circumsporozoite protein (CSP) has long been a vaccine candidate of interest. CSP is the major component of surface proteins on sporozoites, forming a dense coat on the parasite during this stage of the life cycle (18, 19). CSP is composed of the following three regions: (i) an N terminus that binds heparin sulfate proteoglycans, (ii) a four amino acid repeat region, and (iii) a C terminus that contains a thrombospondin-like type I repeat (TSR) domain. The central repeat region contains the immunodominant B cell epitope (20, 21). The C terminus contains the TSR, T cell epitopes, as well as B cell epitopes (22, 23). The N terminus region is also of importance, as it has been shown to be involved in liver attachment (24) and interacts with liver cells through heparin sulfate; antibodies against this region have been shown to be inhibitory in a sporozoite invasion assay (25). As for overall structure, it has been shown that CSP forms a long, flexible, rod-like superhelix composed of regular β-turns (26, 27). However, this structure undergoes conformational changes during the parasite’s life cycle (28). As referenced above, CSP interacts with heparan sulfate proteoglycans on the surface of hepatocytes during the invasion process (29–31). This interaction is contingent on processing of the N terminus and the subsequent conformation change (28). Further, a synthetic peptide corresponding to L86 to G100 blocks salivary gland invasion, showing the biological importance of the N-terminal domain (32). The processing of CSP by a parasite cysteine protease, and subsequent cleavage, is specifically associated with the decision between productive invasion and cell transversal (19, 31), as the proteolytic cleavage of CSP regulates the switch to an open adhesive confirmation, whereas the masking of this domain maintains the sporozoite in a migratory state (18). Native CSP on the surface of sporozoites appears as a glycosylphosphatidylinositol (GPI)-anchored, flexible rod-like protein (26, 27). GPI lipidation is crucial for proper expression of CSP; lipidation is a multistep process, which culminates in the GPI anchor being added to the C-terminal region by a highly specific transamidase (33).

It has been previously shown that in the context of DNA vaccines, the removal of the CSP GPI anchor improves immunogenicity; differences between plasmodial and mammalian GPI signal sequences make CSP a poor substrate for mammalian transamidase, which in turn causes retention of CSP within internal cell organelles (34). This knowledge of the importance of all three domains of CSP, as well as the importance of its confirmation and processing, shaped our decisions for construct design. To that end, five synDNA constructs were designed with variations hypothesized to provide relevant expression and folding or for testing the importance of specific domains of CSP for inducing protection (Table 1).

**TABLE 1:** Construct design

Construct name	Modifications	Justification (reference[s])
3D7	IgE LS	IgE leader sequence improves expression of synDNA constructs (57, 58)
GPI1	IgE LS, mammalian GPI	The use of a mammalian GPI anchor signal sequence was postulated to improve processing in mammalian versus plasmodial cells, thus reducing retention in internal cell organelles (33).
ΔGPI	IgE LS, no GPI	Removal of plasmodial GPI anchor has been shown to increase solubility and secretion and subsequent immunogenicity of CSP DNA vaccines (33)
TM	IgE LS, mammalian transmembrane (TM) domain substituted for native GPI	The use of a mammalian TM domain was postulated to improve processing in mammalian versus plasmodial cells, thus reducing retention in internal cell organelles; CD8 TM region was chosen, as it is adaptable and widely used in chimeric antigen receptor engineering (35)
DD2	IgE LS, dimer of N-terminal sequences from 3D7 and DD2 P. falciparum strains	Testing importance of N-terminal focused immunity versus other regions (i.e., repeat region)

The construct “3D7” contains the unadulterated full-length native CSP sequence from the P. falciparum 3D7 strain. The construct “GPI1” contains the CSP sequence from the P. falciparum 3D7 strain, with a mammalian GPI anchor substituted in for the native protozoan GPI anchor. The construct “ΔGPI” contains the native CSP sequence from the P. falciparum 3D7 strain without a GPI anchor. The construct “TM” contains the CSP sequence from the P. falciparum 3D7 strain with a mammalian transmembrane domain substituted in for the native protozoan GPI anchor. The CD8 TM region was chosen because it is widely and successfully used in chimeric antigen receptor engineering (35). 3D7, GPI1, ΔGPI, and TM were designed to assess whether different cell membrane attachment approaches would modulate antigen production and availability and subsequent immunogenicity. The construct “DD2_3D7” contains the N terminus region of CSP from the P. falciparum DD2 strain, linked to the N terminus region of CSP from the P. falciparum 3D7 strain. DD2 was specifically designed to assess the importance of immunity against the N-terminal domain in CSP-targeted immunity.

These five variations on CSP tested soluble versus secreted forms, different cell membrane attachment approaches, oligomerization, and the importance of the domains of CSP in immunization and challenge studies. Mice immunized with 3D7, dGPI, GPI1, or TM all developed antibody responses against both recombinant CSP (rCSP) as well as the NANP7 peptide, whereas mice immunized with DD2 had a negligible antibody response. Additionally, all 5 constructs induced interferon gamma (IFN-γ) cellular responses with ΔGPI being the highest inducer. Finally, mice immunized with ΔGPI had the highest inhibition of liver-stage infection in a murine challenge model. The vaccine with the highest immunogenicity and most robust protective efficacy was ΔGPI.

Additionally, based on previous work in the lab demonstrating the ability of DNA-launched nanoparticles to assemble *in vivo* (7), we developed mimics of the leading CSP vaccines, RTS,S and R21 (both recombinant protein-based polyvalent vaccines), and tested them side by side with our other DNA vaccines. synDNA RTS,S (dRTS,S) and synDNA R21 (dR21) displayed similar immunogenicity and protective efficacy to their protein counterparts. Both dRTS,S and dR21 elicited anti-CSP antibodies, and only dRTS,S elicited anti-HBsAg antibodies, where dR21 did not, recapitulating what has been seen in prior studies (10). Additionally, both dRTS,S- and dR21-vaccinated mice produced IFN-γ in response to stimulation with CSP antigen. Consequently, dRTS,S and dR21, as well as ΔGPI, demonstrated high protective efficacy against a rigorous infectious mosquito bite murine malaria challenge.

Thus, we observe that a uniquely designed synthetic DNA vaccine, ΔGPI, as well as genetically encoded RTS,S and R21 form polyvalent protein structures which elicit a robust anti-CSP antibody response as well as a T cell response, which was associated with protection from *Plasmodium* infection. Additional study of this approach appears warranted based on our demonstration of robust immunogenicity in the putative correlates of protection for malaria infection, specifically the high-titer antibody response, high antigen-specific IFN-γ production, and the sterilizing immunity demonstrated in challenge.

## RESULTS

### synDNA CSP vaccine construct design and *in vitro* expression.

**FIG 1:**
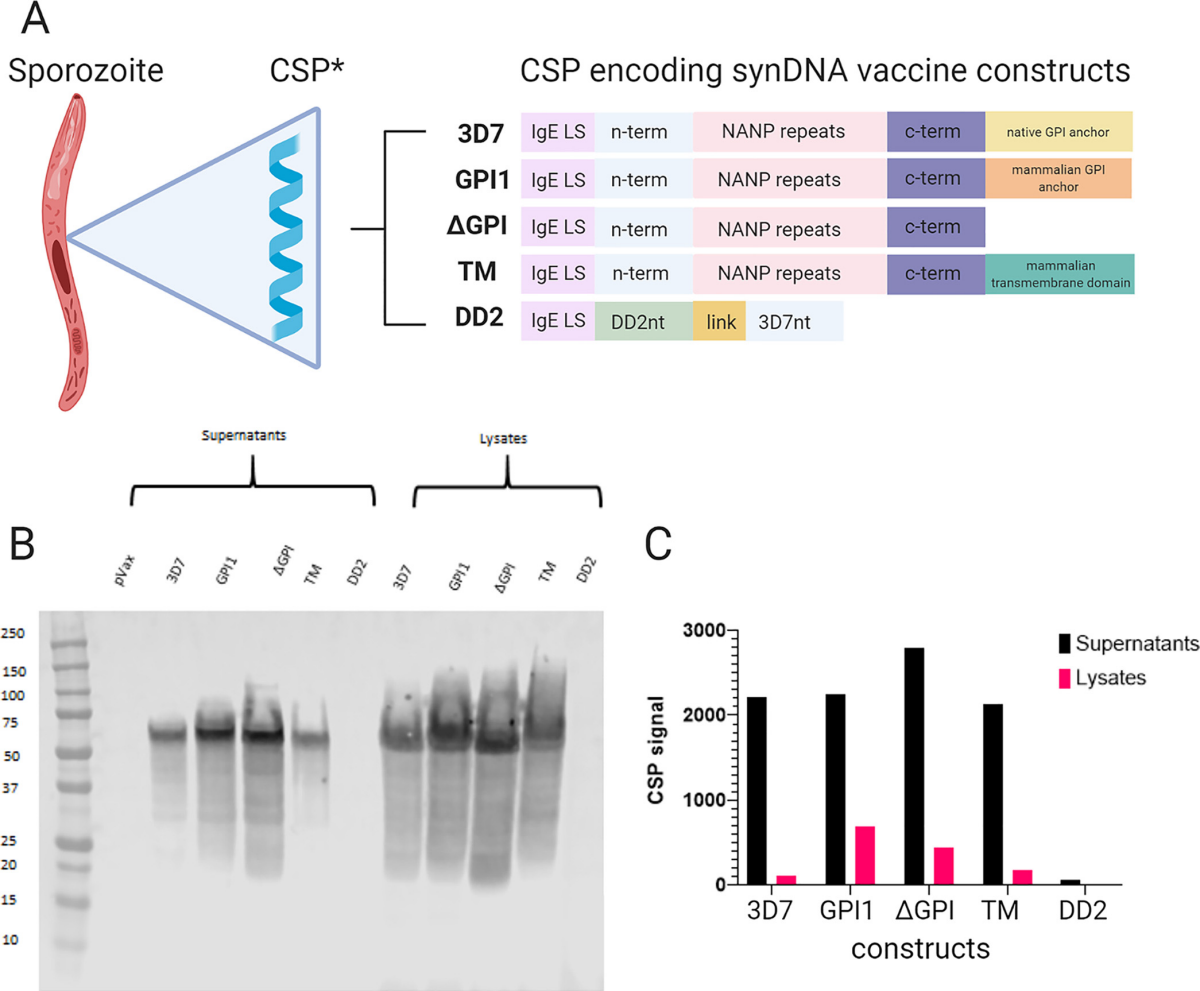
DNA vaccine construct design and *in vitro* expression. (A) Schematic diagram of Plasmodium falciparum gene inserts used to generate the codon-optimized DNA vaccine constructs. The schematic details leader sequence (IgE) and gene insert. *, This does not represent the true structure of CSP and is merely a graphic. (B) *In vitro* expression of vaccine constructs in 293T cells via Western blotting, anti-CSP monoclonal Ab MAb 311 used as probe. (C) Quantified CSP signal from panel B.

Five synDNA constructs were designed with variations hypothesized to increase immunogenicity. All constructs target Plasmodium falciparum. Each construct was synthesized and then inserted into a pVax backbone with an added IgE leader sequence as described (6). The construct “3D7” contains the full-length native CSP sequence from the P. falciparum 3D7 strain. The construct “GPI1” contains the CSP sequence from the P. falciparum 3D7 strain, with a mammalian GPI anchor substituted in for the native protozoan GPI anchor. The construct “ΔGPI” contains the native CSP sequence from the P. falciparum 3D7 strain without a GPI anchor. The construct “TM” contains the CSP sequence from the P. falciparum 3D7 strain with a mammalian transmembrane domain substituted in for the native protozoan GPI anchor. The construct “DD2_3D7” contains the N terminus region of CSP from the P. falciparum DD2 strain, linked to the N terminus region of CSP from the P. falciparum 3D7 strain. These five variations on CSP tested soluble versus secreted forms, different cell membrane attachment approaches, and the importance of domains of CSP in immunization and challenge studies. *In vitro* expression of each construct was verified by Western blotting. Because the detection antibody (Ab) used was directed against the repeat region, the construct DD2 would not be readily detected by this method (Fig. 1B). The synDNA vaccine construct ΔGPI exhibited the highest expression in the supernatants collected from transfected cells (Fig. 1C). This may indicate a superior ability to be secreted from transfected myocytes *in vivo* and thus superior immune activation, though further study is needed.

### synDNA CSP vaccine constructs elicit a robust immune response.

**FIG 2:**
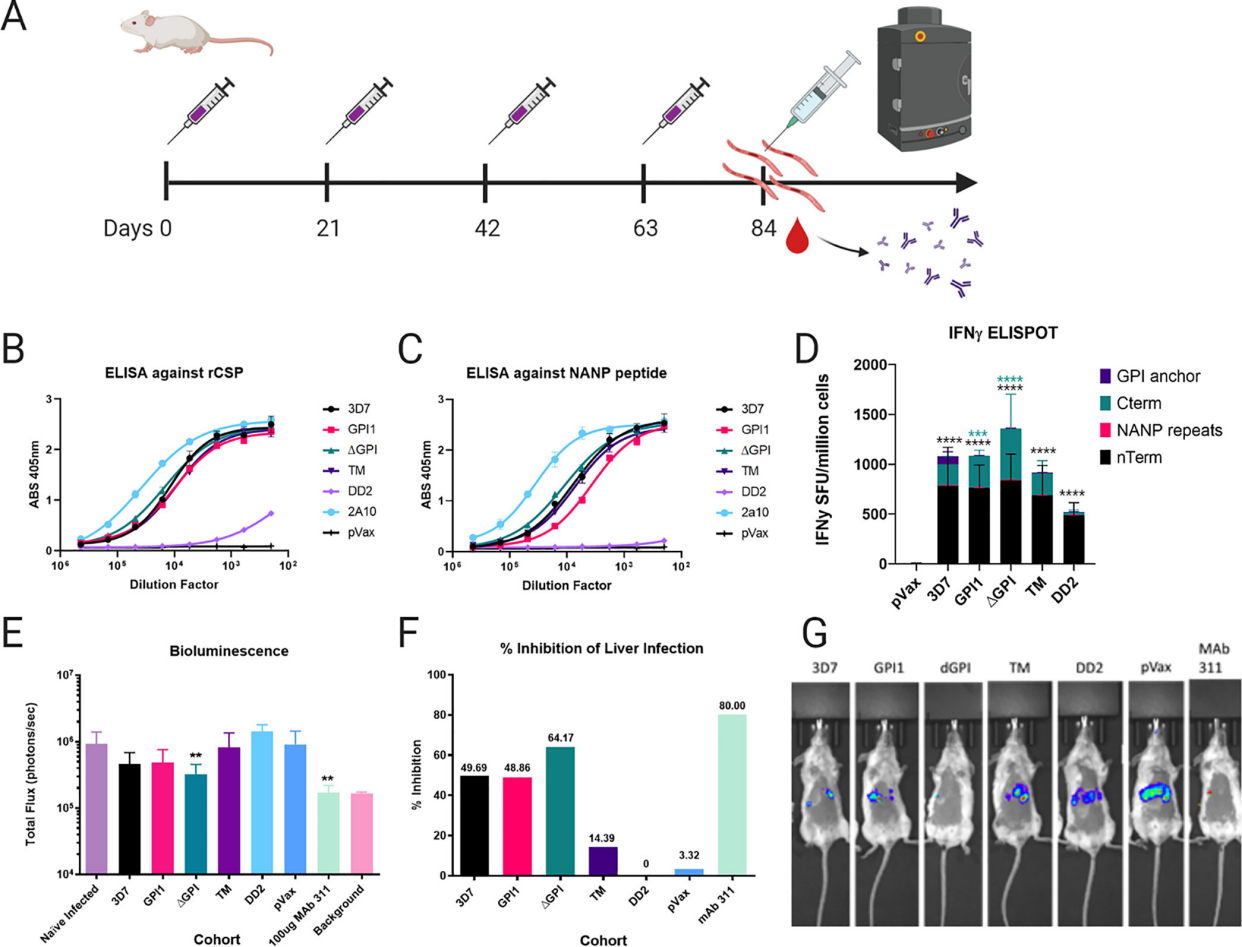
synDNA CSP vaccine constructs elicit a robust immune response and are protective against i.v. sporozoite P. falciparum challenge. (A) Experiment layout. Mice were immunized four times, 3 weeks apart and challenged with 250 sporozoites i.v. 3 weeks after the last immunization. Serum samples were collected prior to challenge for Ab analysis. Liver parasite burden was measured by IVIS. In a separate experiment, mice received the same treatment, and splenocytes were harvested for immune cell analysis as in panel D. (B and C) ELISA’s of pooled sera for each cohort. (B) Tested sera against recombinant CSP; (C) tested sera against the NANP peptide. Pooled sera was initially diluted 1:200, and 3-fold serial dilutions were made afterwards. Monoclonal antibody 2A10 was used as a positive control. (D) The P. falciparum CSP antigen-specific cellular immune response induced by the indicated DNA vaccine measured by IFN-γ ELISPOT. Cells were stimulated for 18 h with peptide pools encompassing the entire protein. A two-way ANOVA with Dunnett’s multiple-comparison test was used to analyze the data. See Table S2 in the supplemental material for details. (E) Graphical representation of luminescence data. Bar graph of mean luminescence for each group and results of Mann-Whitney tests comparing vaccinated groups to sham-inoculated infected mice. Sham-inoculated mice were immunized with pVax, the empty plasmid backbone. Both Ab 311 and ΔGPI demonstrate statistically significant differences compared to sham-inoculated mice (**, *P* < 0.05). (F) Inhibition of liver infection as expressed as a function of relative infection compared to that of sham-inoculated mice. Mice immunized with ΔGPI have the highest inhibition of liver infection (64.17%), while 311 treatment demonstrates an 80% inhibition of liver-stage infection. (G) Representative IVIS images for each experimental group.

To assess the immunogenicity of each unique construct, groups of five BALB/c mice were immunized with 25 μg of vaccine four times 3 weeks apart (weeks 0, 3, 6, and 9) (Fig. 2A). pVax was included as an empty vector (negative) control. Serum samples were collected to assess the antibody response. Previous work has shown that antibodies are of critical importance for targeting CSP, particularly the NANP region (36). The serum samples from immunized mice were used as a primary antibody to probe enzyme-linked immunosorbent assay (ELISA) plates, which had been coated with either recombinant CSP or the NANP peptide alone. The monoclonal antibody 2A10 (37) was used as a control. Mice immunized with 3D7, ΔGPI, GPI1, or TM all developed antibody responses against both rCSP and the NANP peptide. Mice immunized with DD2 induced a negligible antibody response against both rCSP and the NANP peptide, and those immunized with the empty vector control had no antibody response (Fig. 2B and C). In a parallel experiment, splenocytes were collected 1 week after final immunization for immune analysis, and antigen-specific cytokine production was assessed by IFN-γ enzyme-linked immunosorbent spot assay (ELISPOT). All 5 constructs induced IFN-γ cellular responses, with ΔGPI being the most potent. 3D7, GPI1, and TM induced lower but still robust levels of IFN-γ secreting cells, and DD2 induced readily detectable, but the lowest, levels of IFN-γ-secreting cells (Fig. 2D). The bulk of the IFN-γ response is directed against the N terminus of CSP, followed in reactivity by the C terminus. The NANP repeat region induced a low level of IFN-γ. 3D7, the only construct designed to contain a P. falciparum GPI anchor, did elicit a response to the GPI anchor (Fig. 2D).

### synDNA CSP vaccine constructs are protective against i.v. sporozoite P. falciparum challenge.

Groups of five BALB/c mice were immunized with 25 μg of vaccine four times, 3 weeks apart (weeks 0, 3, 6, and 9) as in Fig. 2A. Two weeks after the last boost, mice were inoculated with 250 sporozoites intravenously (i.v.). Immunized and nonimmunized BALB/c mice were challenged with Plasmodium berghei sporozoites expressing both the P. falciparum circumsporozoite protein (CSP) and luciferase. Forty-two hours after intravenous injection of 250 sporozoites, mice were intraperitoneally injected with 100 μl of d-luciferin (30 mg/ml), anesthetized, and liver luminescence was measured with the Perkin Elmer IVIS Spectrum imaging system to assay liver loads. Inhibition of liver infection is expressed as a percent reduction of luminescence in the vaccinated mice, compared to that of the negative control, the pVax sham-vaccinated mice. Mice immunized with ΔGPI have the highest inhibition of liver infection (64.17%) out of the immunized groups, while the positive control, which was 100 μg of the monoclonal antibody 311 (MAb 311) (36) delivered 16 h before challenge showed an 80% inhibition of liver-stage infection (Fig. 2E to G). All immunized groups with the exception of DD2 exhibited some level of protection from liver-stage infection (Fig. 2E to G). We postulate that the lack of protection seen in DD2 immunized mice may be due to the lack of an anti-CSP antibody response, as seen in Fig. 2B and C, though the exact mechanisms of protection certainly merit further study. ΔGPI showed significant vaccine-induced protective immunity in this challenge model (Fig. 2E to G).

### DNA-encoded CSP polyvalent vaccines.

**FIG 3:**
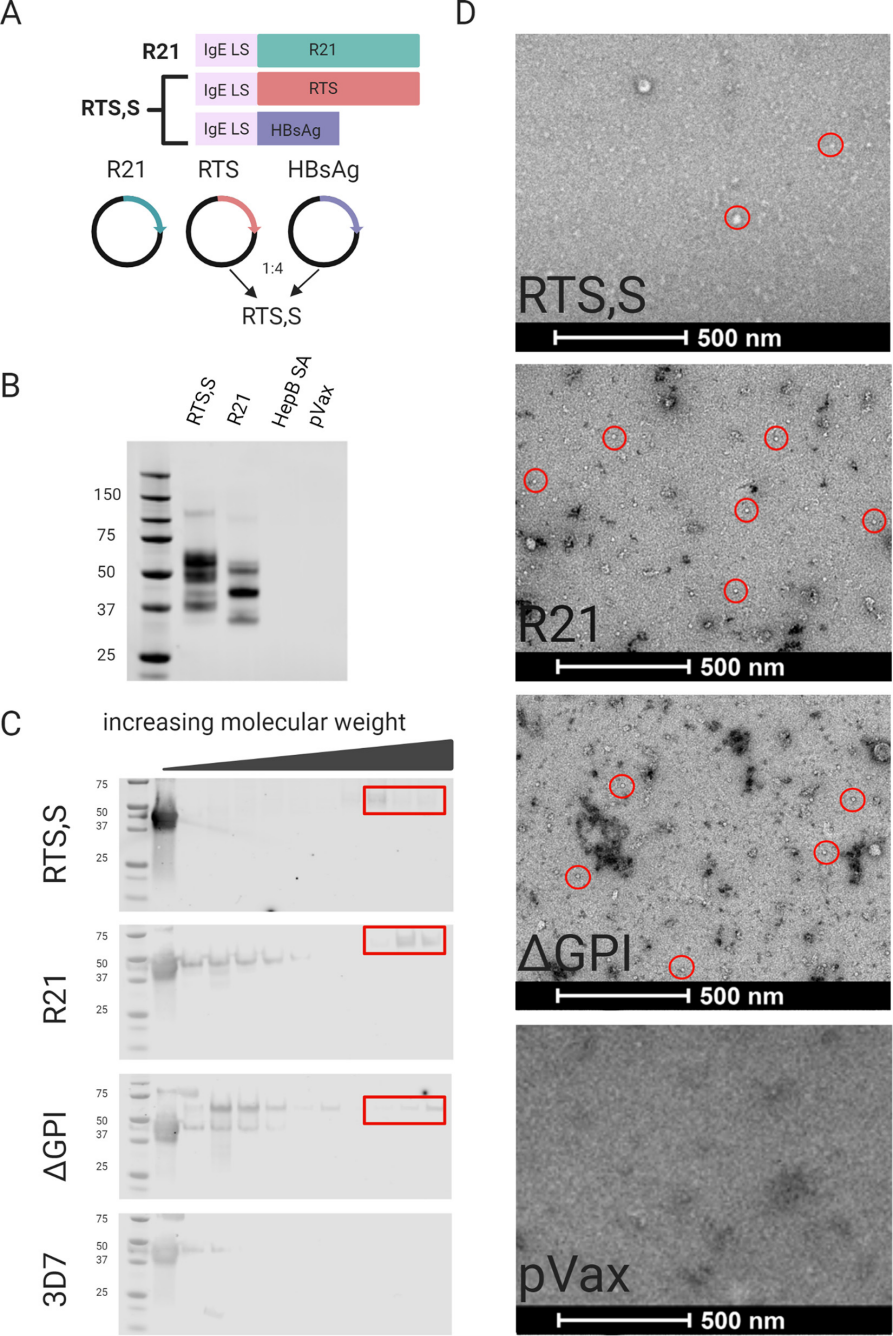
The development of benchmark CSP control vaccines. (A) Schematic diagram of gene inserts used to generate the DNA vaccine constructs. The schematic details leader sequence (IgE) and gene insert as well as ratio of delivery. (B) Western blot of construct expression; supernatants from transfected 293T cells were probed with the MAb 311 (anti-CSP). (C) Discontinuous sucrose gradient fractions probed for CSP. The high-molecular-weight fractions denoted in red boxes were combined and imaged by negative staining electron microscopy as shown in panel D. (D) Particle formation of vaccines *in vitro*. Examples of particles are encircled (not exhaustive, i.e., not all particles are circled). Sham-inoculated cells (pVax) are included as a negative control.

We were encouraged by these initial challenge results and wanted to compare them to the important standards in the field (RTS,S and R21), which are more complex vaccine formulations, both of which form polyvalent particles. However, research acquisition of the protein forms was limited. Thus, to compare more polyvalent forms, we moved to develop genetically encoded DNA vaccines in the form of dR21 and dRTS,S. The sequences for R21 (38) and RTS,S (39) were retrieved and then modified to include changes in RNA and codon bias, as well as the addition of an efficient IgE leader sequence, to generated dR21 and dRTS,S. The final constructs were inserted into a pVax backbone (Fig. 3A). The dR21 construct is delivered *in vivo* as is, as it is a singular antigen fusion between the hepatitis B surface antigen (HBsAg) and CSP. To assemble a mimic of RTS,S, we designed two constructs, one encoding HBsAg and a second RTS construct. The final plasmids were mixed at a 1:4 ratio of RTS to HBsAg (Fig. 3A) to mimic its production as a final protein particle antigen (39).

Expression of each construct was studied by Western blotting of supernatants collected from transfected cells using the anti-CSP monoclonal antibody 311 for detection. dR21 and dRTS,S both expressed *in vitro* (Fig. 3B). To elucidate whether dR21 and dRTS,S were forming nanoparticles similarly to their protein formulation counterparts, supernatants from transfected cells were run on a discontinuous sucrose gradient and separated by molecular weight via ultracentrifugation for 24 h. The resulting gradient fractions were analyzed by Western blotting for CSP expression (Fig. 3C). We observed protein bands with high molecular weight from supernatants collected from both dRTS,S and dR21 transfected cells, which may correspond to higher-order structures such as polyvalent particles. We also observed CSP in high-molecular-weight fractions of supernatants collected from ΔGPI transfected cells, suggesting that this vaccine construct is also capable of forming multimeric aggregates similar to dRTS,S and dR21. This phenomenon was not observed in supernatants collected from 3D7 transfected cells and thus may be a contributing factor behind the superior protection observed in Fig. 2. To evaluate the biophysical properties of the high-molecular-weight fractions, which contained CSP (Fig. 3C), we analyzed relevant fractions with negative stain electron microscopy (nsEM). Protein aggregates, which may be nanoparticles ranging in diameter from 20 nm to 50 nm, were observed in the high-molecular-weight fractions from dRTS,S, dR21, and ΔGPI transfected cells (Fig. 3D).

**FIG 4:**
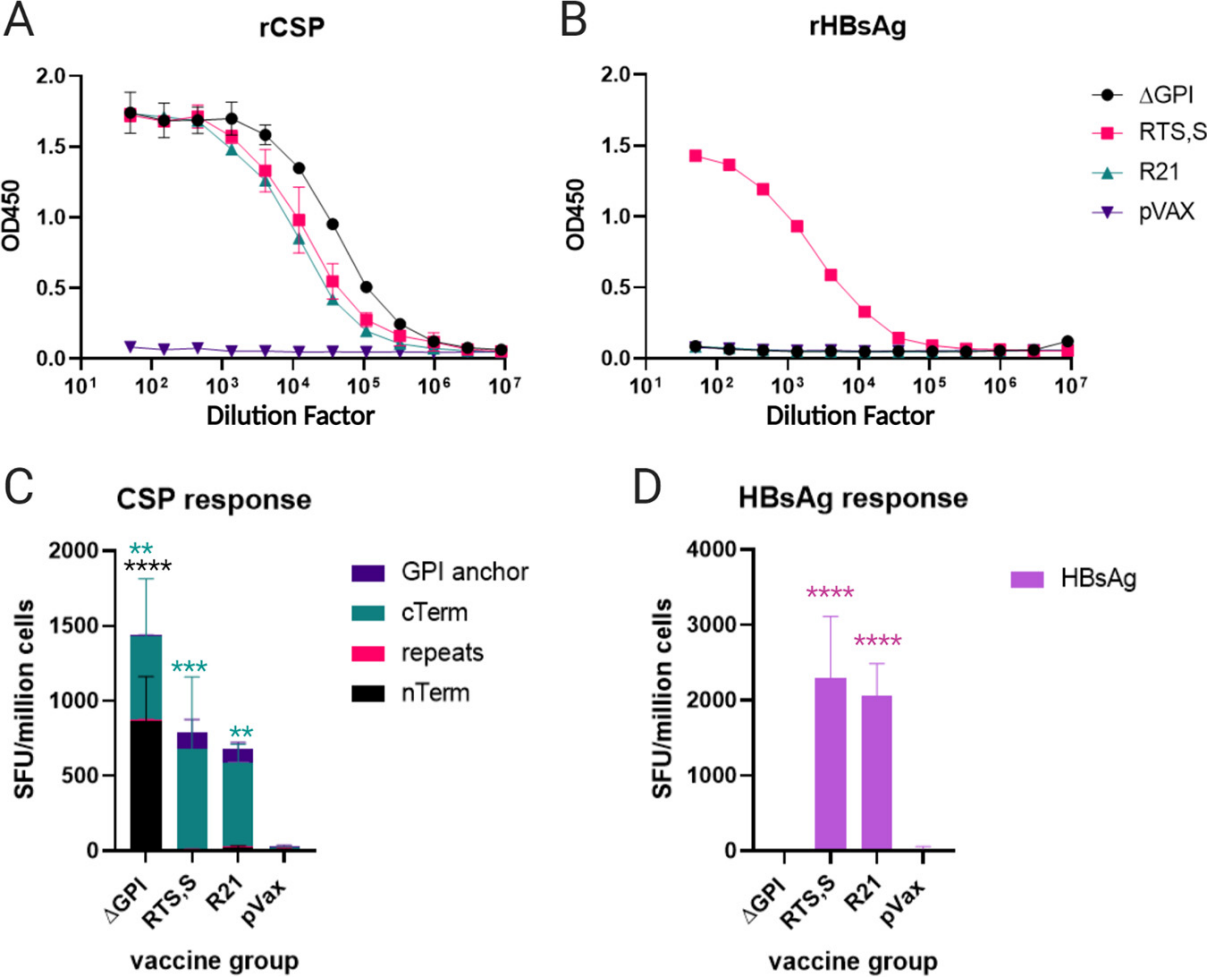
synDNA CSP constructs elicit a robust response. Mice were immunized four times, 3 weeks apart. Serum samples and splenocytes were collected 3 weeks after the last immunization for immune analysis. (A) ELISAs to assess rCSP binding by Ab elicited after final vaccination. (B) ELISAs to assess HBsAg binding by Ab elicited after final vaccination. (C) The P. falciparum CSP antigen-specific cellular immune response induced by the indicated DNA vaccine measured by IFN-γ ELISPOT. Cells were stimulated for 18 h with peptide pools encompassing the entire protein. (D) The HBsAg-specific cellular immune response induced by the indicated DNA vaccine measured by IFN-γ ELISPOT. Cells were stimulated for 18 h with peptide pools encompassing the entire protein. (C and D) A two-way ANOVA with Dunnett’s multiple-comparison test was used to compare each vaccination group to the sham-vaccinated control. Statistical details can be found in Table S3 in the supplemental material.

Both dRTS,S and dR21 induced a potent IFN-γ cellular response. Using separate peptide pools for mapping, we observed that the majority of the CSP response to dRTS,S and dR21 was directed at the C terminus region of CSP. Although the cellular response does target CSP, a majority of the vaccine-induced cellular response was specific to HBsAg but not to CSP (Fig. 4D). In contrast, immunization with ΔGPI only elicits an IFN-γ response against the CSP protein and at a higher magnitude than dR21 and dRTS,S (Fig. 4C and D). However, this focusing of the cellular response onto the carrier protein did not impair the antibody response, as immunization with both dRTS,S and dR21 elicited robust anti-rCSP antibody titers. Comparison of the responses demonstrated that immunization with ΔGPI resulted in slightly higher Ab responses against recombinant CSP than dR21 and dRTS,S (Fig. 4A). Neither ΔGPI nor dR21 elicited an anti-HBsAg antibody response, whereas dRTS,S did (Fig. 4B). The higher molar ratio of HBsAg in dRTS,S may be responsible for this observation; however, further study of these differences could generate additional insight.

### Polyvalent CSP constructs elicit a robust Ab response and are protective against a rigorous mosquito bite challenge model.

**FIG 5:**
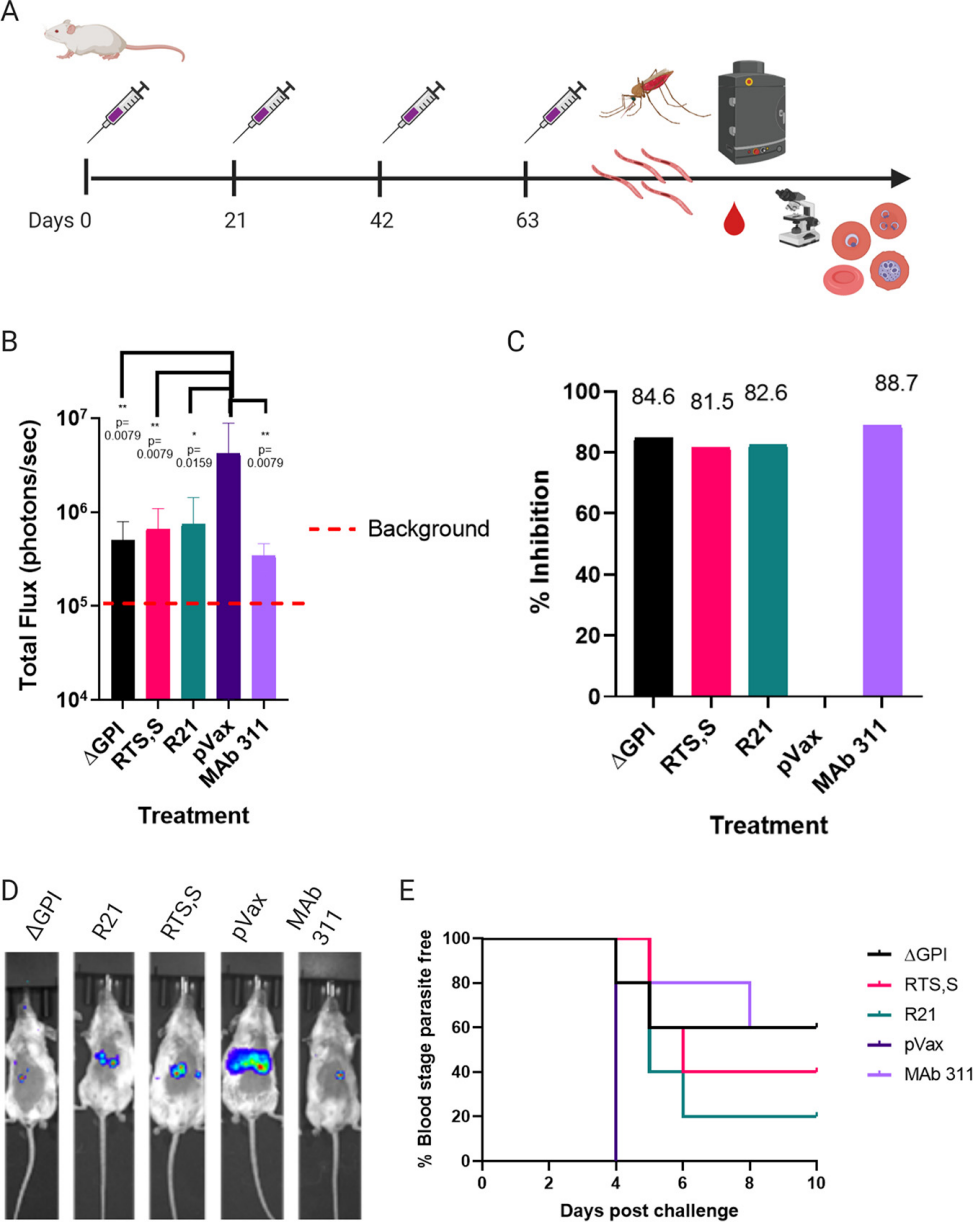
synDNA CSP constructs are protective in challenge. (A) Experimental layout is as follows. mice were immunized four times, 3 weeks apart and challenged 3 weeks after the last immunization by infected mosquito bite. Liver parasite burden was assessed by IVIS. Blood parasite burden was assessed by daily blood smears. (B) Graphical representation of luminescence data. Bars indicate the means and standard deviations. Bar graph of means and results of Mann-Whitney tests comparing groups to sham inoculated mice as a negative control. (C) Inhibition of liver infection expressed as a function of relative infection compared to that of sham-inoculated mice. (D) Representative IVIS images from each group. (E) Percentage blood-stage parasite-free mice as assessed by blood smears each day postchallenge.

ΔGPI, the most potent construct from the initial CSP DNA vaccine study and challenge experiment, and the construct which demonstrated the formation of high-molecular-weight protein aggregates along with dRTS,S and R21, were evaluated in a more rigorous mosquito bite malaria challenge model (40). Groups of five BALB/c mice were immunized with 25 μg of vaccine four times, 3 weeks apart (weeks 0, 3, 6, and 9). Three weeks after the last immunization, the mice were challenged with Plasmodium berghei transgenic parasites, which express the full-length Plasmodium falciparum circumsporozoite protein (*Pf*CSP) as well as luciferase as a reporter for liver parasite load. This parasite is denoted from this point on as *PbPf*Luc. A cage of Anopheles stephensi mosquitoes, 20 days after blood feeding on *PbPf*Luc-infected mice was found to be 90% infected with *PbPf*Luc. Based on this calculation that 90% of the mosquitos in the cage were infected, 5 mosquitoes were required to challenge mice to ensure robust infection by mosquito bites as previously described (41). Mice were anesthetized with 2% Avertin prior to challenge. Mosquitoes were allowed to feed on the animals for ∼10 min. After feeding, the number of mosquitoes positive for a blood meal was determined (Fig. 5A).

Forty-two hours after challenge, mice were intraperitoneally injected with 100 μl of d-luciferin (30 mg/ml), anesthetized, and liver luminescence was measured by using the Perkin Elmer IVIS Spectrum imaging system to assay liver parasite loads. Inhibition of liver infection is expressed as percent reduction of luminescence in the vaccinated mice compared to that of the negative control, the pVax sham-vaccinated mice. Mice immunized with ΔGPI have the highest inhibition of liver infection observed in vaccinated groups (84.6%), while the positive control, 100 μg of the MAb 311, demonstrates an 88.7% inhibition of liver-stage infection in this model (Fig. 5B to D). dRTS,S and dR21 demonstrated overall potent protection of 81.5% and 82.6% inhibition, respectively (Fig. 5B to D). Beginning on day 4 postinfection, blood smears were taken to evaluate protection from blood-stage (BS) parasitemia. All mice immunized with the empty vector control, pVax, succumbed into blood-stage infection by day 4. Of the five mice immunized with dRTS,S, two mice developed blood-stage parasitemia by day 5 and a third on day 6. Of the five mice immunized with dR21, one developed blood-stage parasitemia at day 4, a total of three by day 5, and 4/5 mice had developed parasitemia by day 6 postinfection. Of the mice immunized with ΔGPI, one fell ill by day 4 and a second by day 5, leaving 3/5 mice with sterile protection from blood-stage parasitemia. Finally, of the mice treated with 100 μg of the MAb 311 as a positive control, one developed BS parasitemia by day 4, and a second developed BS parasitemia by day 8, leaving 3/5 mice with sterile protection from blood-stage parasitemia (Fig. 5E) and serving as a robust control. This is the first demonstration that genetically encoded dRTS,S or dR21 can drive protection, similar to prior reports of these immunogens as protein-based vaccines. Furthermore, in this preliminary report, we showed that ΔGPI induced protection, which was comparable to that of the genetically encoded dRTS,S and dR21 as well as the monoclonal antibody-positive control.

### Conclusions.

CSP has long been a focus in malaria vaccine research, and for good reason, as anti-CSP antibodies are one of the predominant correlates of protection for malaria infection (36). CSP is composed of an N-terminal domain containing a conserved proteolytic cleavage site, a central repeat region, and a C-terminal domain (18). CSP undergoes significant conformational change during the parasite’s migration from the mosquito salivary gland to the mammalian liver. When first entering the bloodstream, CSP is in a folded conformation on the surface of the sporozoite. As the parasite reaches the liver, CSP undergoes proteolytic cleavage, which has been shown to be a critical requirement for hepatocyte invasion (19). In addition, it is known that antibody binding to sporozoites abolishes their motility (42) and induces a cytotoxic effect (43) that neutralizes infectivity. Consequently, antibodies targeting CSP have the potential to strongly inhibit sporozoite invasion of hepatocytes and can thus be protective against disease (44).

RTS,S is a recombinant protein-based vaccine comprised of a fragment of CSP containing a section of the repeat region, and the T cell epitopes of the C terminus attached to the hepatitis B surface antigen protein and delivered with additional HBsAg to encourage the formation of virus-like particles in yeast, which are then harvested and administered with an adjuvant to humans to generate a T cell and antibody response. In contrast, while the R21 vaccine is also a recombinant protein-based vaccine containing the same NANP repeats and T cell epitopes of the C terminus as RTS,S, it is able to form particles in yeast without additional HBsAg, meaning that a higher proportion of antigen seen by the immune system will be *Plasmodium* antigen rather than hepatitis B antigen. RTS,S, when formulated with adjuvant, induces antigen-specific humoral and CD4 T cell cellular responses in BALB/c mice (45) and in humans (46–49). R21, when formulated with adjuvant or delivered with a TRAP (thrombospondin-related adhesion protein) based viral vector prime-boost, also demonstrated a robust humoral and cellular response (10). Genetically encoded RTS,S and R21 surrogates were highly immunogenic and protective in a rigorous malaria challenge model and performed similarly to their protein counterparts (10, 45–48, 50). However, the majority of the IFN-γ response elicited by dRTS,S and dR21 was directed against the HBsAg rather than against CSP. In contrast, our primary CSP candidate vaccine, ΔGPI, elicited a cellular response to only CSP, not to HBsAg, thus the response appears more focused toward the CSP antigen targeted by the vaccine.

This study demonstrates the potential for a synDNA vaccine targeting CSP to be highly immunogenic and efficacious. We show that a synthetic DNA vaccine targeting the circumsporozoite protein of Plasmodium falciparum can induce a high-titer anti-CSP antibody response, as well as a robust cellular response producing IFN-γ in response to stimulation with CSP antigen. This immunogenicity elicited by the ΔGPI CSP vaccine resulted in protection from infection in multiple models of murine malaria, including an i.v. sporozoite challenge and a rigorous infected mosquito bite challenge. Additionally, this study is the first to show that synDNA mimics of the leading CSP vaccine candidates RTS,S and R21 generate strong humoral and cellular responses, resulting in protection from infection in an infected mosquito bite challenge model. An interesting future study would be to directly compare protein formulated and adjuvanted RTS,S and R21 to these synDNA mimics of RTS,S and R21, which are delivered as DNA by electroporation (EP) which is self adjuvanting, rather than coformulated with the RTS,S protein mixed with the unique AS01 adjuvant. It has been reported that the damage-associated molecular patterns (DAMPs) caused by electroporation (51) may act as a natural adjuvant, and thus this comparison study will provide direct information regarding the two approaches’ immune induction and protective impact.

Nanoparticle vaccines have become a focus in recent years. Nanoparticles, ordered structures with dimensions in the range of 1 to 1,000 nm, can function as both a delivery system and/or immune potentiators (52, 53). Nanoparticles, which have a comparable size to pathogens, are taken up efficiently by antigen-presenting cells (APCs) (52). Further, the display of antigen in a repetitive array mimics the surface of a pathogen (i.e., as CSP densely coats the surface of sporozoites), and this allows for enhancement of innate immune activation, improved drainage and retention in the lymph node, stronger engagement with B cell receptors, and consequently augmented T cell help to B cells (53). Within the context of prophylactic malaria vaccines, nanoparticle vaccines have been shown to drive broader humoral responses, a balanced Th1/Th2 cytokine profile, and robust germinal center formulation (54). Malaria protein targeting nanoparticles have also been shown to increase Ab titers and increase antigen-specific plasmablasts, circulating memory B cells, and plasma cells in the bone marrow, as well as inducing antigen-specific circulating T follicular helper cells (55). There is precedent for synDNA launched nanoparticle vaccines spontaneously self-assembling *in vivo* and driving stronger humoral responses than monomeric DNA vaccines (7). Thus, we postulate that our synDNA vaccine mimics of the nanoparticle forming RTS,S and R21 vaccines, as well as our novel synDNA vaccine “ΔGPI,” all of which form high molecular weight protein aggregates *in vitro*, may promote enhanced trafficking to lymph nodes, robust germinal center formation, and consequent increases in anti-CSP antibodies compared to non-nanoparticle-forming vaccines.

Further experimentation is needed to elucidate how the high-molecular-weight protein aggregates formed by ΔGPI differ from RTS,S and R21 HBsAg-based particles as well as their mechanisms of immune activation, which may differ from a more conventional synDNA vaccine. We believe this platform of *in vivo* launched polyvalent nanoparticle vaccines targeting CSP has the potential to deliver high-level protection from malaria infection, in a temperature stable and cost-effective manner, which makes it particularly well suited to use in low-resource settings.

## MATERIALS AND METHODS

### Cell lines and transfection.

To probe for *in vitro* expression of the vaccine constructs, the Expi293F transfection kit (Thermo Fisher) was used for all transfections. Expi293F cells were maintained in Expi293 expression medium for passages, and cells were incubated in 8% CO_2_ conditions on an orbital shaker at 37°C. Briefly, 1 day prior to transfection, 2 × 10^5^ Expi293F cells at 95% viability or greater were plated in expression medium. DNA plasmids were added to Opti-MEM medium separately from ExpiFectamine transfection reagent. After a 5-min incubation period, DNA and ExpiFectamine were complexed during a 20-min incubation period. Subsequently, the DNA plasmid complex was added to Expi293F cells in suspension. Eighteen hours after the addition of DNA, transfection enhancers were added according to the manufacturer’s instructions. After three to 5 days, cell supernatants and lysates were collected for further studies.

### Western blotting.

To detect vaccine construct expression in transfection supernatants and lysates, 12 μl of sample was run on 4 to 12% Bis-Tris gel (Thermo Fisher) in morpholineethanesulfonic acid (MES) buffer. Samples were boiled and reduced before being run. Upon completion of the gel, contents of the gel were transferred to a nitrocellulose membrane via the iBlot 2 transfer system (Thermo Fisher). Upon transfer completion, the membrane was blocked using Intercept blocking buffer (LI-COR) for 1 h at room temperature. After blocking, the membranes were probed with the anti-CSP human MAb 311 at a 1:1,000 dilution in blocking buffer at 4°C overnight. The following day, a fluorescently labeled anti-human secondary antibody was added to the membrane, formulated in Intercept blocking buffer, SDS, phosphate-buffered saline (PBS), and Tween 20 for a 1-h incubation. Following secondary incubation, the membrane was washed with PBS + 0.1% Tween 20 four times and was subsequently imaged using the LI-COR Odyssey CLx.

### Ultracentrifugation and gradient fraction collection.

In order to assess the structural formation of vaccine constructs, transfectant supernatants were collected and filtered through a 0.45-μm filter to remove cell debris. Then an Amicon Ultra-15 10k filter was used to concentrate 15 ml of sample with a 4,000 × *g* spin for approximately 15 to 40 min. Concentrated protein (1 g) was loaded onto a 5 ml 10 to 50% discontinuous sucrose gradient (50 mM Tris, pH 8, 150 mM NaCl, 2 mM EDTA, 50 mM NaF, 5 mM sodium pyrophosphate, 10 to 50% sucrose) and ultracentrifuged (39,800 rpm) for 24 h at 4°C. Fractions (250 μl) were collected and stored at −20°C.

### Electron microscopy.

Fractions were collected from the density gradient analysis and dialyzes into PBS overnight using Slide-A-Lyzer Mini dialysis devices (Thermo Fisher) before being concentrated via an Amicon Ultra-0.5 ml 10k filter. Three microliters of each sample was applied to a thin carbon grid that was glow discharged for 30 s at 30 mAmps current using Pelco easiGlow glow discharger. Three microliters of freshly made solution of 2% uranyl acetate was used to stain each sample twice on the grid with 1 min of incubation time. Excess stain and sample were removed by carefully blotting the grid at the edge with a Whatman filter, and the grid was allowed to dry until imaged. Transmission electron microscopy (TEM) micrographs were collected using a Tecnai T12 TEM microscope operated at 100 KeV, and the images were recorded at ×20 magnification on a Gatan 4K complementary metal oxide semiconductor (CMOS) camera.

### ELISA.

For binding detection of CSP in transfection supernatants as well as quantification of serum antibody titers, MaxiSorp 96-well plates or half-area plates (Thermo Fisher) were coated overnight with 1 μg/ml of recombinant CSP (courtesy of MVI/PATH) at 4°C. The next day, each plate was washed with phosphate buffered saline + 0.01% Tween 20 (PBS-T) four times (4×). Plates were then blocked with 5% milk in PBS for 2 h at room temperature (RT). Upon completion of blocking, plates were washed again, and samples diluted in 1% newborn calf serum (NCS) in PBS-T were transferred onto the plates for a 2-h incubation at RT. Following sample incubation, plates were washed, and goat anti-mouse or anti-human heavy- and light-chain horseradish peroxidase (HRP)-conjugated secondary was diluted to 1:10,000 and transferred onto plates for a 1-h incubation at RT. After secondary incubation, plates were washed and developed using SIGMAFAST OPD (Sigma-Aldrich) for 10 min and then stopped with sulfuric acid. The BioTek Synergy 2 plate reader was used to read plates at 450 nm. Data were exported to Microsoft Excel and analyzed using GraphPad Prism 8.

### Animal studies and immunizations.

Six- to eight-week-old female BALB/cJ mice were purchased from the Jackson Laboratory, and each animal study was repeated twice to ensure reliability of data. Animal experiments were conducted under protocol number 201236 approved by the Wistar Institutional Animal Care and Use Committee (IACUC). All animals were housed in the Wistar Institute Animal Facility, with the exception of challenge studies, which were performed at the Johns Hopkins Bloomberg School of Public Health. Mice were immunized with 25 μg of DNA in sterile water intramuscularly in the tibialis anterior (TA) muscle of the right leg and subsequently electroporated using the CELLECTRA 3P adaptive electroporation device (Inovio Pharmaceuticals) as previously described (56). Mice were immunized four times, 3 weeks apart for all experiments, including the challenge experiments. One week following each vaccination, blood was collected via submandibular bleed to isolate serum for future experiments. In addition, 1 week after the final immunization, mice were euthanized for splenocyte collection for subsequent assays.

### Sample processing and ELISPOT.

Following euthanasia, spleens were harvested and temporarily stored in R10, consisting of 10% fetal bovine serum (FBS) and 1% penicillin-streptomycin in RPMI 1640 (Invitrogen). Using the Stomacher 80 tissue stomacher, spleens were homogenized for 1 min before filtering through a 40-μm strainer. The cell mixture was centrifuged for 10 min at 1,200 rpm and subsequently resuspended in ACK lysis buffer (Gibco) for a 5-min period. Cells were washed with PBS prior to centrifugation and resuspension in 20 ml R10 medium for counting on a Countess II (Invitrogen).

To assess antigen-specific interferon gamma (IFN-γ) production, mouse IFN-γ ELISPOT PLUS (Mabtech) plates were used according to the protocol provided by the manufacturer. Briefly, plates were washed using sterile PBS followed by blocking with 200 μl per well of R10. Plates were seeded with 200,000 cells in 100 μl R10 in triplicate. Cells were stimulated with peptide pools of 15 mers overlapping by 11 amino acids spanning the entire vaccine antigen at a final concentration of 5 μg/ml per peptide. R10 and concanavalin A were used as negative and positive controls, respectively. After an 18-h incubation in 5% CO_2_ conditions at 37°C, plates were developed according to the protocol provided by the manufacturer. After developing, the CTL ImmunoSpot S6 Universal Analyzer (Cellular Technology Limited) was used to scan and count plates. Data analysis was performed using GraphPad Prism 8. For statistical analysis of ELISPOT data, a two-way analysis of variance (ANOVA) with Dunnett’s multiple comparisons was used to compare each vaccination group to the sham-inoculated control.

### i.v. sporozoite challenge.

Challenge study was performed at Johns Hopkins under IACUC number MO16H35. Each mouse was immunized via intramuscular injection followed by electroporation on weeks 0, 3, 6, and 9. On week 11 of the experiment, mice were challenged with Plasmodium berghei transgenic parasites that express the full-length Plasmodium falciparum circumsporozoite protein (*Pf*CSP) as well as luciferase to report liver parasite load as described (41). This parasite is denoted from this point on as *PbPf*Luc. Briefly, the 311 one hundred-microgram cohort was injected with 100 μg of human MAb 311 16 h prior to challenge, and age-matched all other mice; this cohort served as a control for protection. Forty-two hours after intravenous injection of 250 sporozoites, mice were intraperitoneally injected with 100 μl of d-luciferin (30 mg/ml), anesthetized, and liver luminescence was measured with the Perkin Elmer IVIS Spectrum imaging system to assay liver loads. Mouse background luminescence was determined by first removing mouse abdominal hair with Nair cream, anesthetizing the mice in an isoflurane chamber, and once immobilized placing them in an IVIS spectrum imager to evaluate bioluminescence by measuring the radiance for 5 min from the abdomen of uninfected mice injected with luciferin. Mann-Whitney tests were used to compare luminescence between immunized groups and the sham-inoculated negative control, considering the sample size and the lack of evidence for normal distribution of the data.

### Infectious mosquito bite challenge.

Challenge study was performed at Johns Hopkins under IACUC number MO16H35. Each mouse was immunized via intramuscular injection followed by electroporation on weeks 0, 3, 6, and 9. Serum was isolated from each mouse via retro orbital bleeding 3 days prior to challenge. The 311 one hundred-microgram cohort was injected with 100 μg of human MAb 311 16 h prior to challenge, and age-matched all other mice; this cohort served as a control for protection. On week 12 of the experiment, mice were challenged with *PbPf*Luc via infectious mosquito bite. A cage of Anopheles stephensi mosquitoes, 20 days after blood feeding on *PbPf*Luc-infected mice was determined to be 90% infected with *PbPf*Luc. Based on this calculation, it was determined that 5 mosquitoes were needed to challenge mice with infected mosquito bites as previously described (41). Briefly, mice were anesthetized with 2% Avertin prior to challenge. Mosquitoes were allowed to feed on mice for ∼10 min. After feeding, the number of mosquitoes positive for a blood meal was determined. Forty-two hours after mosquito bite challenge, liver parasite load was measured using the Perkin Elmer IVIS Spectrum imaging system. Mice were injected with 100 μl of d-Luciferin (30 mg/ml), anesthetized with isoflurane, and imaged with the IVIS Spectrum to measure bioluminescence expressed by the transgenic parasites. Mouse background luminescence was determined by first removing mouse abdominal hair with Nair cream, anesthetizing the mice in an isoflurane chamber, and, once immobilized, placing them in an IVIS Spectrum imager to evaluate bioluminescence by measuring the radiance for 5 min from the abdomen of uninfected mice injected with luciferin. Mann-Whitney tests were used to compare luminescence between immunized groups and the sham-inoculated negative control, considering the sample size and the lack of evidence for normal distribution of the data. Blood smears were taken beginning on day 4 postinfection to evaluate parasitemia. A positive result was considered an endpoint. Mice were euthanized upon confirmation of blood stage parasitemia.
