# Supplementary material for: Strategic Variants of CSP Delivered as SynDNA Vaccines Demonstrate Heterogeneity of Immunogenicity and Protection from Plasmodium Infection in a Murine Model
Source: Infect Immun. 2021 Sep 16;89(10):e00728-20. doi: 10.1128/IAI.00728-20 (PMC8445182; doi:10.1128/IAI.00728-20)
Supplement: Supplemental file 1 — Supplemental material. Download IAI.00728-20-s0001.pdf, PDF file, 0.08 MB [file iai.00728-20-s0001.pdf]

Supplemental Table 1: SynDNA Vaccine Construct Sequences

Construct name	DNA Sequence
3D7	GGATCCGCCG CCACCATGGA TTGGACATGG ATTCTGTTTC TGGTCGCTGC CGCCACACGG<br>GTGCATAGTC TGTTTCAGGA GTATCAGTGC TACGGGTCTT CCTCCAACAC CAGGGTGCTG<br>AACGAGCTGA ATTACGATAA CGCCGGCACA AATCTGTATA ACGAGCTGGA GATGAACTAC<br>TATGGCAAGC AGGAGAACTG GTACTCTCTG AAGAAGAACT CTAGGAGCCT GGGCGAGAAC<br>GACGATGGCA ACAATGAGGA CAACGAGAAG CTGAGAAAGC CCAAGCACAA GAAGCTGAAG<br>CAGCCTGCCG ACGGCAACCC CGATCCTAAC GCCAATCCTA ACGTGGACCC AAATGCCAAC<br>CCCAACGTGG ACCCCAACGC CAATCCAAAC GTGGACCCCA ATGCCAACCC TAATGCCAAT<br>CCTAATGCCA ATCCAAACGC TAACCCTAAT GCCAATCCCA ATGCCAACCC AAACGCCAAC<br>CCTAATGCCA ACCCCAACGC CAACCCAAAT GCTAACCCTA ATGCCAACCC AAATGCAAAT<br>CCTAACGCAA ATCCAAACGC CAATCCTAAT GCCAACCCCA ATGCTAATCC AAACGCCAAC<br>CCAAACGTGG ACCCCAACGC TAATCCTAAC GCCAATCCAA ACGCCAATCC CAACGCCAAC<br>CCTAACGCTA ACCCTAACGC TAACCCCAAC GCCAATCCTA ACGCTAACCC AAATGCTAAT<br>CCCAATGCTA ACCCAAATGC AAACCCTAAC GCTAATCCAA ATGCTAACCC AAATGCCAAC<br>CCTAACGCTA ATCCCAATGC TAATCCCAAT GCAAACCCTA ACGCCAATCC CAACAAGAAC<br>AATCAGGGCA ACGGCCAGGG CCACAATATG CCAAACGACC CCAATCGGAA CGTGGATGAG<br>AATGCCAACG CCAATTCTGC CGTGAAGAAC AATAACAATG AGGAGCCAAG CGATAAGCAC<br>ATCAAGGAGT ACCTGAATAA GATCCAGAAC TCCCTGTCTA CCGAGTGGAG CCCTTGCTCC<br>GTGACATGTG GCAACGGCAT CCAGGTGCGC ATCAAGCCTG GCAGCGCCAA CAAGCCAAAG<br>GACGAGCTGG ATTATGCCAA CGACATCGAG AAGAAGATCT GCAAGATGGA GAAGTGTAGC<br>TCCGTGTTTA ACGTGGTCAA TAGTAGCATC GGGCTGATTA TGGTCCTGTC CTTTCTGTTC<br>CTGAATTGAT AACTCGAG
GPI1	GGATCCGCCG CCACCATGGA TTGGACTTGG ATTCTGTTTC TGGTCGCCGC CGCTACACGG<br>GTGCATTCTC TGTTTCAGGA GTATCAGTGC TATGGGTCAT CAAGCAACAC CAGGGTGCTG<br>AACGAGCTGA ATTACGATAA CGCCGGCACA AATCTGTATA ACGAGCTGGA GATGAACTAC<br>TATGGCAAGC AGGAGAACTG GTACTCCCTG AAGAAGAACT CTAGGAGCCT GGGCGAGAAC<br>GACGATGGCA ACAATGAGGA CAACGAGAAG CTGAGAAAGC CAAAGCACAA GAAGCTGAAG<br>CAGCCAGCAG ACGGAAACCC AGATCCCAAC GCCAATCCCA ACGTGGACCC TAATGCCAAC<br>CCAAACGTGG ACCCCAACGC CAATCCTAAC GTGGACCCAA ATGCCAACCC CAATGCCAAT<br>CCAAATGCCA ACCCTAACGC CAACCCCAAT GCCAACCCAA ACGCCAACCC TAATGCTAAC<br>CCCAATGCCA ATCCCAATGC CAACCCTAAT GCAAACCCTA ACGCCAATCC TAACGCCAAC<br>CCTAATGCCA ATCCTAACGC TAATCCTAAT GCCAACCCTA ATGCGAATCC TAACGCTAAC<br>CCTAACGTGG ACCCCAATGC TAATCCAAAT GCTAACCCCA ACGCTAATCC AAACGCCAAT<br>CCCAACGCTA ACCCCAACGC AAACCCCAAC GCTAACCCTA ATGCTAACCC AAATGCTAAC<br>CCTAATGCTA ATCCTAACGC AAACCCAAAT GCAAACCCAA ATGCAAATCC TAATGCTAAC<br>CCCAACGCAA ATCCTAATGC CAATCCCAAC GCTAATCCCA ACGCCAATCC AAACAAGAAC<br>AATCAGGGCA ACGGCCAGGG CCACAATATG CCTAACGACC CAAATCGGAA CGTGGATGAG<br>AATGCCAACG CCAATTCCGC CGTGAAGAAC AATAACAATG AGGAGCCTTC TGATAAGCAC<br>ATCAAGGAGT ACCTGAATAA GATCCAGAAC TCCCTGTCTA CCGAGTGGAG CCCATGCTCC<br>GTGACATGTG GCAACGGCAT CCAGGTGCGC ATCAAGCCCG GCTCTGCCAA CAAGCCTAAG<br>GACGAGCTGG ATTATGCCAA CGACATCGAG AAGAAGATCT GCAAGATGGA GAAGTGTAGC<br>TCCGTGTCTG CCGCAAGCTG CCCACAGCCC CCTACATTTC TGCTGCCCCT GCTGCTGTTT<br>CTGGCTCTGA CTGTCGCAAG ACCAAGGTGG AGATGATAAC TCGAG
dGPI	GGATCCGCCG CCACCATGGA TTGGACTTGG ATTCTGTTTC TGGTCGCCGC AGCAACTAGA<br>GTGCATAGCC TGTTTCAGGA GTATCAGTGT TACGGGTCAT CATCTAACAC CAGGGTGCTG<br>AACGAGCTGA ATTACGATAA CGCCGGCACA AATCTGTATA ACGAGCTGGA GATGAACTAC<br>TATGGCAAGC AGGAGAACTG GTACAGCCTG AAGAAGAACA GCAGGTCCCT GGGCGAGAAC<br>GACGATGGCA ACAATGAGGA CAACGAGAAG CTGAGAAAGC CCAAGCACAA GAAGCTGAAG<br>CAGCCTGCCG ACGGCAACCC CGATCCTAAC GCCAATCCTA ACGTGGACCC AAATGCCAAC<br>CCCAACGTGG ACCCCAACGC CAATCCAAAC GTGGACCCCA ATGCCAACCC TAATGCCAAT<br>CCTAATGCCA ATCCAAACGC TAACCCTAAT GCCAATCCCA ATGCCAACCC AAACGCCAAC<br>CCTAATGCCA ACCCCAACGC CAACCCAAAT GCTAACCCTA ATGCCAACCC AAATGCAAAT<br>CCTAACGCAA ATCCAAACGC CAATCCTAAT GCCAACCCCA ATGCTAATCC AAACGCCAAC

	CCAAACGTGG ACCCCAACGC TAATCCTAAC GCCAATCCAA ACGCCAATCC CAACGCCAAC<br>CCTAACGCTA ACCCTAACGC TAACCCCAAC GCCAATCCTA ACGCTAACCC AAATGCTAAT<br>CCCAATGCTA ACCCAAATGC AAACCCTAAC GCTAATCCAA ATGCTAACCC AAATGCCAAC<br>CCTAACGCTA ATCCCAATGC TAATCCCAAT GCAAACCCTA ACGCCAATCC CAACAAGAAC<br>AATCAGGGCA ACGGCCAGGG CCACAATATG CCAAACGACC CCAATCGGAA CGTGGATGAG<br>AATGCCAACG CCAATAGCGC CGTGAAGAAC AATAACAATG AGGAGCCATC CGATAAGCAC<br>ATCAAGGAGT ATCTGAATAA GATCCAGAAC TCTCTGAGCA CCGAGTGGTC CCCTTGCTCT<br>GTGACATGTG GCAACGGCAT CCAGGTGCGC ATCAAGCCTG GCTCCGCCAA CAAGCCAAAG<br>GACGAGCTGG ACTACGCAAA TGATATTGAA AAGAAAATCT GTAAAATGGA AAAGTGCTCC<br>TCAGTGTGAT AACTCGAG
TM	GGATCCGCCG CCACCATGGA TTGGACTTGG ATTCTGTTTC TGGTCGCCGC CGCTACACGA<br>GTGCATTCTC TGTTTCAGGA GTATCAGTGC TATGGAAGCT CATCCAACAC CAGGGTGCTG<br>AACGAGCTGA ATTACGATAA CGCCGGCACA AATCTGTATA ACGAGCTGGA GATGAACTAC<br>TATGGCAAGC AGGAGAACTG GTACTCTCTG AAGAAGAACT CTAGGAGCCT GGGCGAGAAC<br>GACGATGGCA ACAATGAGGA CAACGAGAAG CTGAGAAAGC CCAAGCACAA GAAGCTGAAG<br>CAGCCTGCCG ACGGCAACCC CGATCCTAAC GCCAATCCTA ACGTGGACCC AAATGCCAAC<br>CCCAACGTGG ACCCCAACGC CAATCCAAAC GTGGACCCCA ATGCCAACCC TAATGCCAAT<br>CCTAATGCCA ATCCAAACGC TAACCCTAAT GCCAATCCCA ATGCCAACCC AAACGCCAAC<br>CCTAATGCCA ACCCCAACGC CAACCCAAAT GCTAACCCTA ATGCCAACCC AAATGCAAAT<br>CCTAACGCAA ATCCAAACGC CAATCCTAAT GCCAACCCCA ATGCTAATCC AAACGCCAAC<br>CCAAACGTGG ACCCCAACGC TAATCCTAAC GCCAATCCAA ACGCCAATCC CAACGCCAAC<br>CCTAACGCTA ACCCTAACGC TAACCCCAAC GCCAATCCTA ACGCTAACCC AAATGCTAAT<br>CCCAATGCTA ACCCAAATGC AAACCCTAAC GCTAATCCAA ATGCTAACCC AAATGCCAAC<br>CCTAACGCTA ATCCCAATGC TAATCCCAAT GCAAACCCTA ACGCCAATCC CAACAAGAAC<br>AATCAGGGCA ACGGCCAGGG CCACAATATG CCAAACGACC CCAATCGGAA CGTGGATGAG<br>AATGCCAACG CCAATTCTGC CGTGAAGAAC AATAACAATG AGGAGCCAAG CGACAAGCAC<br>ATCAAGGAGT ATCTGAATAA GATCCAGAAC TCCCTGTCTA CCGAGTGGAG CCCTTGCTCC<br>GTGACATGTG GCAACGGCAT CCAGGTGCGC ATCAAGCCTG GCAGCGCCAA CAAGCCAAAG<br>GACGAGCTGG ATTACGCCAA CGATATCGAG AAGAAGATCT GCAAGATGGA GAAGTGTAGC<br>TCCGTGGACA TCTATATCTG GGCTCCTCTG GCTGGCACCT GCGGCGTCCT GCTGCTGTCC<br>CTGGTCATTA CTCTGTATTG ATAACTCGAG
DD2	GGATCCGCCG CCACCATGGA TTGGACTTGG ATTCTGTTTC TGGTCGCTGC CGCAACTAGA<br>GTGCATTCCC TGTTTCAGGA GTATCAGTGC TATGGGTCAT CAAGCAATAC CCGGGTGCTG<br>AATGAGCTGA ACTATGATAA CGCCGGCACC AATCTGTACA ACGAGCTGGA GATGAACTAT<br>TACGGCAAGC AGGAGAACTG GTACAGCCTG AAGAAGAACT CCAGAAGCCT GGGCGAGAAC<br>GACGATGGCA ATAACGAGGA CAACGAGAAG CTGAGAAAGC CCAAGCACAA GAAGCTGAAG<br>CAGCCTGCCG ATGGCAACCC TGATCCAGGC GGCGGCGGCT CTGGCGGCGG CGGCAGCGGC<br>GGCGGCGGCT CCCTGTTTCA GGAGTACCAG TGTTATGGCT CTAGCTCCAA CACCAGAGTG<br>CTGAACGAGC TGAATTACGA CAACGCCGGC ACAAACCTGT ACAATGAGCT GGAGATGAAC<br>TACTATGGCA AGCAGGAGAA TTGGTACTCC CTGAAGAAGA ATTCTAGATC CCTGGGCGAG<br>AATGACGATG GCAACAATAA CAACGGCGAT AATGGCAGAG AGGGCAAGGA CGAGGATAAG<br>AGGGACGGCA ACAATGAGGA CAACGAGAAA CTGCGGAAGC CTAAGCATAA AAAACTGAAA<br>CAGCCAGGAG ACGGAAATCC AGACCCATGA TAACTCGAG
RTS	GGATCCGCCA CCATGGACTG GACTTGGATT CTGTTCCTGG TCGCTGCCGC AACTAGAGTG<br>CATAGCATGG CTCCCGACCC TAACGCAAAC CCTAACGCAA ACCCTAATGC CAACCCTAAC<br>GCCAATCCTA ATGCCAACCC AAATGCCAAC CCAAACGCCA ATCCAAACGC CAACCCTAAT<br>GCCAATCCCA ATGCCAACCC CAACGCCAAT CCTAACGCCA ACCCCAATGC CAACCCCAAT<br>GCCAATCCTA ACGCCAATCC CAACGCCAAT CCCAACGCCA ACCCAAATGC CAACCCTAAT<br>AAGAACAATC AGGGCAATGG CCAGGGCCAC AACATGCCCA ACGACCCAAA TCGGAACGTG<br>GACGAGAACG CCAACGCCAA TTCTGCCGTG AAGAACAATA ATAACGAGGA GCCCAGCGAC<br>AAGCACATCA AGGAGTATCT GAACAAGATC CAGAACTCCC TGAGCACCGA GTGGAGCCCT<br>TGTTCTGTGA CCTGTGGCAA CGGCATCCAG GTGCGCATCA AGCCTGGCAG CGCCAACAAG<br>CCAAAGGATG AGCTGGACTA TGCCAACGAC ATCGAGAAGA AGATCTGCAA GATGGAGAAG<br>TGCTCCAGCG TGTTTAACGT GGTGAATTCT AGCATCGGCC TGGGCCCCGT GACAAATATG<br>GAGAACATCA CCTCCGGCTT TCTGGGCCCA CTGCTGGTGC TGCAGGCCGG CTTCTTTCTG<br>CTGACCCGCA TCCTGACCAT CCCTCAGTCT CTGGACTCCT GGTGGACCTC CCTGAATTTT

	CTGGGCGGCA GCCCCGTGTG CCTGGGCCAG AACTCCCAGT CTCCTACATC CAACCACAGC<br>CCCACCTCTT GTCCACCCAT CTGTCCTGGC TATCGCTGGA TGTGCCTGAG AAGGTTCATC<br>ATCTTTCTGT TCATCCTGCT GCTGTGCCTG ATCTTTCTGC TGGTGCTGCT GGACTATCAG<br>GGCATGCTGC CAGTGTGCCC TCTGATCCCT GGCAGCACAA CCACCAATAC AGGCCCATGC<br>AAGACATGTA CAACCCCTGC CCAGGGCAAC AGCATGTTTC CCAGCTGTTG TTGCACCAAG<br>CCAACAGACG GCAACTGCAC CTGCATCCCA ATCCCTAGCA GCTGGGCCTT CGCCAAGTAC<br>CTGTGGGAGT GGGCCTCTGT GAGGTTCTCC TGGCTGTCTC TGCTGGTGCC ATTCGTGCAG<br>TGGTTTGTGG GCCTGAGCCC AACAGTGTGG CTGAGCGCCA TCTGGATGAT GTGGTATTGG<br>GGCCCTAGCC TGTACTCAAT CGTGAGCCCT TTCATTCCCC TGCTGCCAAT CTTCTTTTGC<br>CTGTGGGTCT ACATTTGATA ACTCGAG
S	GGATCCGCCA CCATGGACTG GACATGGATT CTGTTTCTGG TCGCTGCTGC TACAAGAGTG<br>CACAGTATGG AGAACATCAC CTCCGGCTTC CTGGGCCCCC TGCTGGTGCT GCAGGCCGGC<br>TTCTTTCTGC TGACCAGAAT CCTGACCATC CCTCAGTCCC TGGATAGCTG GTGGACATCC<br>CTGAATTTCC TGGGCGGCTC CCCCGTGTGC CTGGGCCAGA ACTCTCAGTC CCCCACATCC<br>AACCACAGCC CAACATCCTG TCCTCCAATC TGCCCAGGCT ACAGATGGAT GTGCCTGCGG<br>AGATTCATCA TCTTCCTGTT CATCCTGCTG CTGTGCCTGA TCTTTCTGCT GGTGCTGCTG<br>GATTACCAGG GCATGCTGCC AGTGTGCCCA CTGATCCCAG GCTCCACCAC AACCAATACA<br>GGCCCATGCA AGACATGTAC CACACCTGCC CAGGGCAATT CCATGTTTCC AAGCTGCTGT<br>TGTACCAAGC CTACCGACGG CAACTGTACA TGCATCCCTA TCCCAAGCTC TTGGGCCTTC<br>GCCAAGTACC TGTGGGAGTG GGCCTCCGTG AGATTTTCTT GGCTGTCCCT GCTGGTGCCA<br>TTTGTGCAGT GGTTTGTGGG CCTGTCCCCA ACAGTGTGGC TGTCTGCCAT CTGGATGATG<br>TGGTACTGGG GCCCAAGCCT GTATTCAATC GTGTCCCCCT TCATTCCCCT GCTGCCAATC<br>TTTTTCTGTC TGTGGGTCTA CATCTGATAA CTCGAG
R21	GGATCCGCCA CCATGGACTG GACATGGATT CTGTTTCTGG TCGCTGCTGC TACAAGAGTG<br>CACAGTGACC CCAACGCTAA CCCCAATGCC AACCCCAATG CCAACCCTAA TGCCAACCCA<br>AACGCCAATC CCAACGCCAA TCCAAACGCC AACCCAAATG CCAACCCCAA CGCCAACCCC<br>AATGCCAATC CCAATGCCAA CCCAAATGCC AATCCAAATG CCAACCCTAA CGCCAATCCC<br>AACGCCAATC CTAACGCCAA TCCAAACGCC AATCCTAACG CCAACCCTAA CAAGAACAAT<br>CAGGGCAACG GCCAGGGCCA CAATATGCCT AATGACCCCA ATCGCAACGT GGATGAGAAC<br>GCCAATGCCA ACAGCGCCGT GAAGAATAAC AACAATGAGG AGCCTTCTGA CAAGCACATC<br>AAGGAGTATC TGAACAAGAT CCAGAACTCC CTGAGCACCG AGTGGAGCCC TTGCAGCGTG<br>ACATGCGGCA ACGGCATCCA GGTGAGAATC AAGCCTGGCT CCGCCAATAA GCCAAAGGAC<br>GAGCTGGACT ACGCCAACGA CATCGAGAAG AAGATCTGCA AGATGGAGAA GTGCTCTTCC<br>GTGCCTGTGA CCAATATGGA GAACATCACC TCCGGCTTCC TGGGCCCCCT GCTGGTGCTG<br>CAGGCCGGCT TCTTTCTGCT GACCAGAATC CTGACCATCC CTCAGTCCCT GGATAGCTGG<br>TGGACATCCC TGAATTTCCT GGGCGGCTCC CCCGTGTGCC TGGGCCAGAA CTCTCAGTCC<br>CCCACATCCA ACCACAGCCC AACATCCTGT CCTCCAATCT GCCCAGGCTA CAGATGGATG<br>TGCCTGCGGA GATTCATCAT CTTCCTGTTC ATCCTGCTGC TGTGCCTGAT CTTTCTGCTG<br>GTGCTGCTGG ATTACCAGGG CATGCTGCCA GTGTGCCCAC TGATCCCAGG CTCCACCACA<br>ACCAATACAG GCCCATGCAA GACATGTACC ACACCTGCCC AGGGCAATTC CATGTTTCCA<br>AGCTGCTGTT GTACCAAGCC TACCGACGGC AACTGTACAT GCATCCCTAT CCCAAGCTCT<br>TGGGCCTTCG CCAAGTACCT GTGGGAGTGG GCCTCCGTGA GATTTTCTTG GCTGTCCCTG<br>CTGGTGCCAT TTGTGCAGTG GTTTGTGGGC CTGTCCCCAA CAGTGTGGCT GTCTGCCATC<br>TGGATGATGT GGTACTGGGG CCCAAGCCTG TATTCAATCG TGTCCCCCTT CATTCCCCTG<br>CTGCCAATCT TTTTCTGTCT GTGGGTCTAC ATCTGATAAC TCGAG
